# PreS/2-21-Guided siRNA Nanoparticles Target to Inhibit Hepatitis B Virus Infection and Replication

**DOI:** 10.3389/fimmu.2022.856463

**Published:** 2022-04-29

**Authors:** Lixia Gao, Jie Yang, Jutao Feng, Ziying Liu, Ying Dong, Jiangyan Luo, Liangzhentian Yu, Jiamei Wang, Hongying Fan, Weifeng Ma, Tiancai Liu

**Affiliations:** ^1^ Department of Microbiology, School of Public Health, Southern Medical University, Guangzhou, China; ^2^ Department of Hepatobiliary Surgery, The First Affiliated Hospital of Guangzhou Medical University, Guangzhou, China; ^3^ State Key Laboratory of Organ Failure Research, Guangdong Provincial Key Laboratory of Viral Hepatitis Research, Department of Infectious Diseases, Nanfang Hospital, Southern Medical University, Guangzhou, China; ^4^ Institute of Antibody Engineering, School of Laboratory Medicine & Biotechnology, Southern Medical University, Guangzhou, China

**Keywords:** HBV, siRNA, peptide, liposomal nanoparticles, targeting

## Abstract

A viable therapy is needed to overcome the deadlock of the incurable chronic hepatitis B (CHB). The prolonged existence of covalently closed circular DNA (cccDNA) and integrated HBV DNA in the nucleus of hepatocytes is the root cause of CHB. As a result, it is critical to successfully suppress HBV DNA replication and eliminate cccDNA. RNA interference has been proven in recent research to silence the expression of target genes and thereby decrease HBV replication. However, siRNA is susceptible to be degraded by RNA enzymes *in vivo*, making it difficult to deliver successfully and lacking of tissue targeting. To exploit the advantages of siRNA technology while also overcoming its limitations, we designed a new strategy and prepared biomimetic nanoparticles that were directed by PreS/2-21 peptides and precisely loaded HBV siRNA. Experiments on these nanoparticles *in vitro* and *in vivo* revealed that they are tiny, stable, safe and highly targetable, with high inhibitory effects on HBV DNA, pgRNA, cccDNA, HBeAg and HBsAg. PreS/2-21-directed nanoparticles loaded with HBV gene therapy drugs are expected to be promising for the treatment of CHB.

## Introduction

Hepatitis B virus (HBV) is a hepatophilic DNA virus and its persistent infection leads to chronic hepatitis B (CHB). HBV is now infecting over 250 million people globally (1), with over 600,000 people dying each year due to progression to cirrhosis, liver failure and hepatocellular carcinoma (HCC) (2). It is clear that there is still no effective response to hepatitis B virus infection. Despite the availability of a highly effective hepatitis B vaccination, hepatitis B virus infection remains a global public health problem. To improve the condition, a more methodical treatment technique must be identified (3). The nucleoside/nucleotide analogues (NAs) and interferon alpha (IFN-α)/pegylated interferon (PEG-IFN), which are currently utilized to treat chronic HBV infection, both have significant drawbacks. Interferon treatment has a wide range of in individual responses, as well as a slew of adverse side effects and drug resistance. NAs inhibit HBV replication directly by suppressing viral reverse transcription, which are well tolerated, promote viral clearance and adherence to treatment (4), as well. However, NAs are unable to provide a cure because they do not remove covalently closed circular DNA (cccDNA). Therefore, developing an effective NAs hepatitis B therapy is critical.

RNA interference (RNAi) is a gene therapy that can be used to inhibit HBV replication and treat hepatitis B by mediating targeted mRNA degradation or mRNA translation inhibition to silence the expression of target genes in a specific manner (5). Clinical studies with siRNA drugs targeting the Pre-C, Pre-S1, Pre-S2, and X genes of HBV are now underway, with promising results (6, 7). Since all five mRNAs transcribed by HBV contain the X gene sequence, siRNA targeting the X gene can simultaneously inhibit the *in vivo* replication of HBV by inhibiting the formation of pregenomic RNA (pgRNA). Silencing the X gene of HBV fundamentally inhibits the translation of viral antigens and prevents liver injury from T-cell immune responses caused by viral antigen accumulation (8, 9). In this study, RNAi-mediated X gene silencing played an important inhibitory role in HBV replication in hepatocytes. *In vivo*, however, siRNA is susceptible to degradation by RNA enzymes, making it challenging to ensure successful delivery of siRNA. Viral vectors, non-viral vectors and chemically modified siRNAs have all been shown to help with this problem to some extent (10), but given the lack of tissue targeting of nanoparticles (11), further special modifications are needed to achieve active targeting of liposomal nanoparticles and thus increasing drug concentrations in liver tissue.

**Figure 1:**
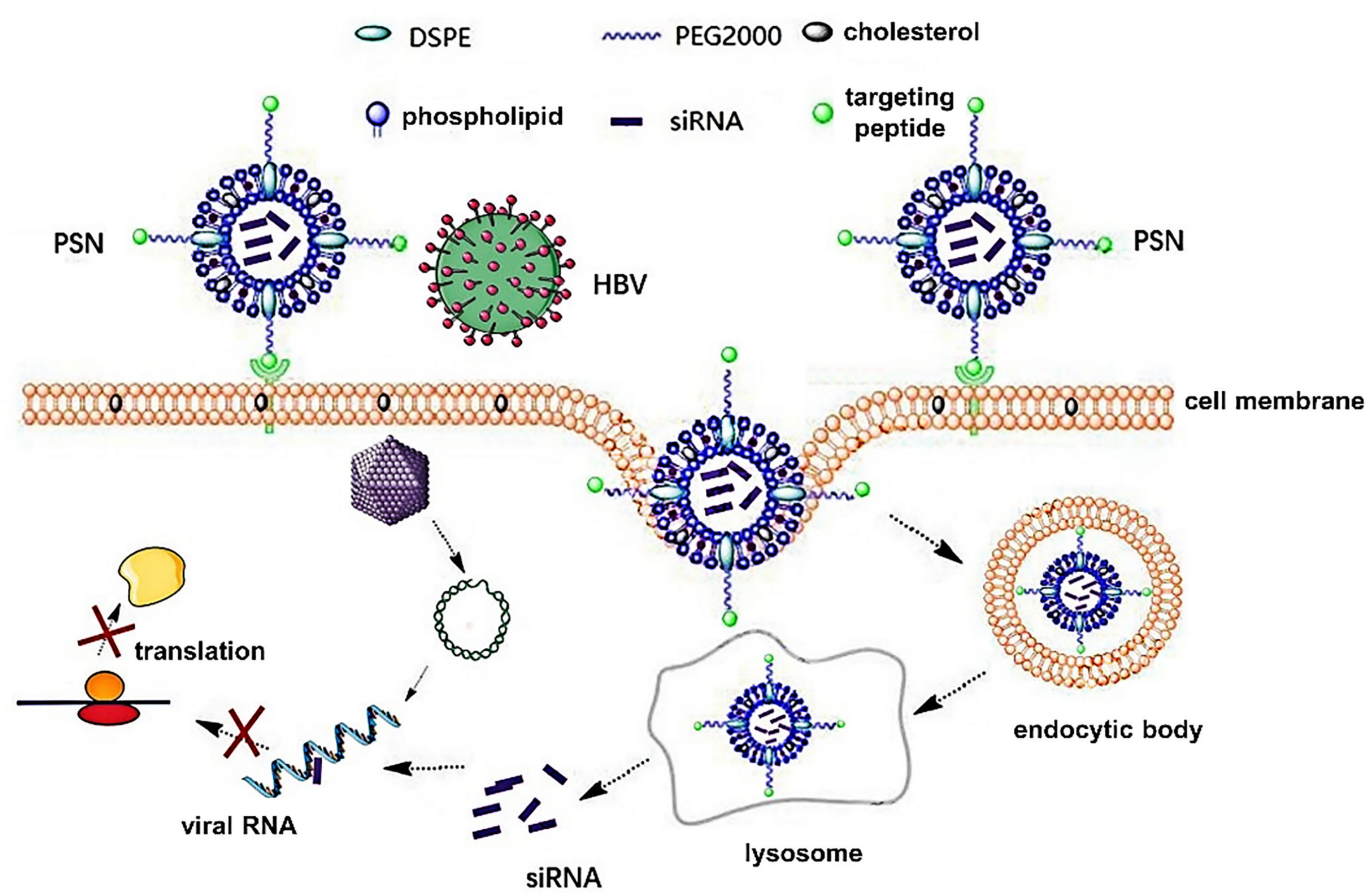
Principles of PreS/2-21-conjugated nanoparticle with siRNA on targeted inhibition of hepatitis B virus.

Studies have confirmed that HBV infects hepatocytes *via* attaching its PreS1 protein to the sodium taurocholate cotransporting polypeptide (NTCP) receptor of the latter (12, 13). Due to the critical function of PreS1 protein in HBV infection, the HBVpreS/2-21^myr^ (the binding region of PreS1) modified long-acting liposomal nanoparticles prepared by Han et al. (14) can specifically deliver fluorescein sodium into hepatocytes with high NTCP expression. Drawing on this, in this paper, PreS/2-21 is employed as a guide peptide, and linked to the surface of siRNA-encapsulated liposomal nanoparticles, which increased the targeting ability of the nanoparticles and competitively inhibited extracellular HBV invasion. Theoretically, the PreS/2-21-modified siRNA nanoparticles (PSN) can exert inhibitory effects on both extracellular and intracellular viruses. On the one hand, PreS/2-21 on the surface of the nanoparticles can target and bind to NTCP receptors on the surface of hepatocytes, acting as both an entry inhibitor to inhibit viral invasion into host cells and a guider for the nanoparticle specific entry into hepatocytes with high NTCP expression. On the other hand, the siRNA drug released *via* endocytosis has the ability to selectively block intracellular viral replication and expression. **Figure 1** depicts the working principles of this system. Through *in vitro* and *in vivo* studies, the anti-HBV viral efficacy, targeting and safety of PSN were comprehensively investigated and evaluated in this work.

## Materials and Methods

### Cells and Animals

HepG2.2.15 cells, HepG2-N6 cells and pHBV1.3 plasmid were donated by Prof. Xiaoyong Zhang from the Department of Infectious Diseases, Nanfang Hospital. In particular, HepG2.2.15 cells, which can stably express HBsAg, HBV DNA and other substances in cell supernatant, are liver tumor cell lines of HepG2 cells with HBV genome integrated on their chromosomes (15), and HepG2-N6 cells are HepG2-derived cell lines that retain the characteristics of polarized hepatocytes, but display the morphology of a single columnar epithelium, enabling routine studies of virus transmission and release (16). In addition, HepG2-N6 cells highly express human NTCP (hNTCP) receptor, which is applicable for HBV targeting research. Mouse macrophage RAW 264.7 was donated by Prof. Longying Zha from the School of Public Health, Southern Medical University; HepG2 cells were kept in our laboratory; HBV/pMD18-T plasmid containing HBV DNA was constructed in our laboratory. HepG2 cells, HepG2-N6 cells, and RAW 264.7 cells were cultured in dulbecco’s modified eagle medium (DMEM) containing 10% serum, and HepG2.2.15 cells were cultured in 1640 medium containing 10% fetal bovine serum, non-essential amino acids, and G418 (200 μg/mL).

Six to eight weeks-old SPF grade C57BL/6J male mice were purchased from the Animal Experiment Centre of Southern Medical University and housed in a sterile SPF grade laminar flow chamber. The mice were given regular mouse chow and ad libitum under controlled conditions of temperature (20-25°C) and humidity (40-45%) using a 12:12 h light/dark cycle. All animal experiments were performed after receiving consent from Southern Medical University’s ethics review committee for animal experimentation.

### Cellular Assay of HBV Inhibition by siRNA

The siRNA-X (1646-1664, GGUCUUACAUAAGAGGACU), siRNA-P (411-429, UCCUGCUGCUAUGCCUCAU), siRNA-C (2019-2039, AAGCCUUAGAGUCUCCUGAGC) against HBV X, P, and C genes respectively were selected by referring literature (17–20) and validated with cellular experiments, and synthesized by Guangzhou RiboBio, Co., Ltd.

The pHBV1.3 plasmid containing 1.3 times of the HBV genome was used for the construction of HBV infection models *in vitro* (21). To produce a cellular model of HBV infection, the pHBV1.3 plasmid was transfected into HepG2 cells using Lipofectamine 3000 (Gibco, Co., Ltd). siRNA was transfected simultaneously with the pHBV1.3 plasmid into HepG2 cells; HepG2.2.15 cells exclusively transfected with siRNA; the control group consisted of cells transfected with non-targeted control siRNA, and three replicate wells were set up for each cell line. After 72 h, the culture supernatants were collected and the levels of HBV DNA and HBsAg in the supernatants were measured. The HBV/pMD18-T plasmid containing HBV DNA was used as the standard of DNA levels. After fold dilution, quantitative real-time polymerase chain reaction (qPCR) was performed according to SYBR Premix Ex TaqTM II instructions (YEASEN, Co., Ltd). The lysis curve was the system default. The standard curve for HBV DNA was plotted using Ct values and the copy number was converted to international unit IU/mL. After centrifuging the supernatant, 50 μL was transferred to a new PCR tube, heated at 100°C for 2 min, then 1 μL was taken into the qPCR system (7), and the level of HBV DNA was calculated using the standard curve. After centrifugation to remove cell debris, the supernatant was obtained and HBsAg ELISA kits (Keygen Biotech, Co., Ltd) were used to detect HBV antigen levels according to the kit instructions.

### Preparation of PSN

The experimental approach described in reference (22) was partially improved to prepare PSN and its controls, PreS/2-21-modified nanoparticles (PN), siRNA nanoparticles (SN), and lipid-like nanoparticles (LLN). TT3 is an organic compound produced the stepwise reaction with addition of propylene diamine, di-tert-butyl dicarbonate, sodium bicarbonate, 1,3,5-benzenetricarbonyl chloride, pyridine, trifluoroacetic acid, ethyl acetate, triethylamine, dodecaldehyde, and triacetyl, and was synthesized by WuXi AppTec. 1,2-distearoyl-sn-glycero-3-phosphocholine (DSPC) was purchased from Shanghai yuanye Bio-Technology, Co., Ltd. 1,2-distearoyl-sn-glycero-3-phosphoethanolamine-N[maleimide(polyethyleneglycol)-2000] (Mal-PEG2000-DSPE) was purchased from Shanghai Aladdin Biochemical Technology, Co., Ltd. The target peptide HBV PreS/2-21 and the lipid were synthesized by ChinaPeptides, Co., Ltd. The sequence of the target peptide is: NH_2_-GTNLSVPNPLGFFPDHQLDP-COOH, synthesized with a stearylation modification at the N-terminal end and a cysteine (Cys) coupled at the C-terminal end. PreS/2-21 was dissolved in PBS solution (pH=7.0), Mal-PEG2000-DSPE was dissolved in N, N-Dimethylformamide (DMF), the two were combined at a molar ratio of Mal-PEG2000-DSPE: PreS/2-21 = 1:1.2 and the dehydration-condensation reaction was carried out slowly at 4°C for 8 h. PreS/2-21-Mal-PEG2000-DSPE (PMD) was obtained by lyophilizing the reaction products, followed by re-solubilizing it with anhydrous ethanol. The extrusion approach was used to synthesize PSN by TT3, cholesterol, DSPC, and PMD in a molar ratio of 50:10:38.5:1.5 (23). The aforementioned ingredients were also partially or completely used to make PN, SN, and LLN (**Table 1**). The above liposomal nanoparticle extrusions were concentrated using an ultrafiltration tube and decontaminated using a 0.22 μm filter membrane.

**Table 1:** The molar ratio of each component in the synthesis of liposomal nanoparticles.

	TT3	DSPC	Cholesterol	PEG2000-DSPE	PMD	siRNA
PSN	50	10	38.5	No	1.5	Yes
PN	50	10	38.5	No	1.5	No
SN	50	10	38.5	1.5	No	Yes
LLN	50	10	38.5	1.5	No	No

### Encapsulation Rate and Characterization of Liposomal Nanoparticles

The concentrations of siRNA were measured using an ultra-micro UV spectrophotometer (Denovix, Co., Ltd) and the number of moles of siRNA was calculated from the volume. The encapsulation rate was calculated as EE% = (moles of siRNA before packaging - moles of siRNA after packaging)/moles of siRNA before packaging × 100%. PSN was naturally dried on aluminum foil and plated with gold, and then the ultrastructural morphology was observed by scanning electron microscope (Hitachi, Co., Ltd). The particle size and zeta potential of liposomal nanoparticles were measured by Zetasizer Nano (Malvern, Co., Ltd): the liposomal nanoparticles diluted with ddH_2_O from 1 μL to 1000 μL were placed in a potentiometric cuvette and then assayed and scanned 100 times in duplicate. The liposomal nanoparticles were stored at 4°C analyzed for the particle size every 5 days, and monitored continuously for 30 days to examine the temporal stability. 10 μL of liposomal nanoparticles were added to 90 μL of PBS containing 10% FBS and shaken at 37°C at 100 rpm/min. The particle size was measured at 0, 3, 6, 12, 24, and 48 h to observe the serum stability. In addition, we monitored the temporal stability of activity by storing the liposomal nanoparticles at 4°C and analyzed the inhibition of HBV DNA by PSN using HepG2.2.15 cells once a week for a month.

### *In Vitro* Safety and Efficacy Testing of Liposomal Nanoparticles

HepG2.2.15, HepG2-N6 and RAW263.7 cells (1×10^4^ cells/well) were grown for 24 h in 96-well plates, then incubated with varying dosages of PSN for 48 h. To assess cytotoxicity, MTT was applied to the samples, and the absorbance values at OD 490 nm were measured to calculate the 50% cytotoxic concentration (CC50) of PSN on different cells. Experiments were performed using the cell models of HepG2.2.15, HepG2 transfected with pHBV1.3 (pHBV1.3-HepG2), and HepG2-N6 cells. Treatment groups consisted of five PSN concentration gradient subgroups, as well as a negative control and a blank control group, each with three replicate wells. The cell supernatant was collected after 72 h of incubation to detect the level of HBV DNA, the inhibition rate of PSN against HBV was calculated, and the half effective concentration (EC50) was estimated by GraphPad Prism 7 software (GraphPad Software, Inc., San Diego, CA, USA). Therapeutic Index (TI) was calculated by the ratio of CC50 against EC50. RAW264 cells were seeded on 6-well plates at a density of 2×10^5^ per well and co-cultured with 25 μg/mL PSN. The cells and culture supernatants were collected respectively after co-cultivation for 12 h, and the expression of cytokines IFN-α, TNF-α, and IL-6 was detected by reverse transcription qPCR (RT-qPCR) and ELISA kits (MULTI SCIENCES(LIANKE) BIOTECH, CO., LTD.).

### *In Vitro* Targeting Assay of PSN on NTCP Receptors

First, the expression of NTCP receptors on the surface of HepG2 cells, HepG2-N6 cells were detected by flow cytometry, fluorescence microscopy, and western blot. The binding ability of PSN to NTCP receptors were detected by flow cytometry and fluorescence microscopy. HepG2 cells and HepG2-N6 cells were plated in six-well plates and incubated for 12 hours followed by a 30 min incubation with PBS or PSN (40 μL), then were stained with FITC-labeled NTCP antibody (ImmunoClone, IC03828F), and an equal amount of cells stained with FITC-labeled isotype control antibody was used as a negative control and analyzed by flow cytometry and fluorescence microscopy. HepG2-N6 cells and HepG2 cells were seeded in six-well plates. After 12-hours incubation, the cells were processed for total protein extraction, concentration determination, and western blot analysis. The protein concentration was measured with Pierce™ BCA Protein Assay Kit (ThermoFisher #23225) following instructions. Western blot was done according to standard methods. The expression level of β-actin was used as an internal control. The primary antibodies used in western blot were NTCP polyclonal antibody (Signalway Antibody Co., United States) and β-actin antibody (Fude Biological Technology Co., Hangzhou, China), and the secondary antibody was horseradish peroxidase-conjugated goat anti-rabbit IgG (Bioworld, United States). Furthermore, liposomal nanoparticles PSN-Cy3 and SN-Cy3 were prepared by Cy3-labeled siRNA-X, and the targeting of PSN-Cy3 to NTCP receptors was detected by HepG2-N6 cells, HepG2 cells and Hela cells. The Cy3-labelled siRNA-X was synthesized by Guangzhou RiboBio, Co., Ltd., and the liposomal nanoparticles were prepared as before, light protection throughout the process. Cells were inoculated into six-well plates and incubated for 12 h, then 40 μL PSN-Cy3 or SN-Cy3 was added and incubated for 30 min protected from light, while PBS-treated cells were used as a negative control. The fluorescence was measured by flow cytometry, observed under a fluorescence microscope and photographed.

### *In Vitro* Inhibition of HBV by PSN

Experiments were performed using the cell models of HepG2.2.15, pHBV1.3-HepG2, and HepG2-N6 cells. Among them, HepG2-N6 cell model was infected with HBV produced in HepG2.2.15 cell culture supernatant and concentrated using PEG8000, and the infection efficiency was verified by western blot. The experiments were divided into five groups: negative control, PSN, PN, SN, and LLN group. Approximately 12 h after the cells were inoculated onto the six-well plates, cells had attained 80% confluency, and 5 nM siRNA-containing nanoparticles or an equivalent amount of nanoparticles were introduced to the cells. Cell supernatants and cells were collected separately after 72 h of incubation, and the expression of HBxAg, pgRNA, HBV DNA and cccDNA were detected using RT-qPCR or qPCR. The primer sequences are shown in **Table S1**. The levels of HBsAg and HBeAg in cell supernatants were detected by ELISA kits (Keygen Biotech, Co., Ltd). The cells were processed for protein extraction, concentration determination, and western blot analysis. Antibody to hepatitis B core antigen (anti-HBc, a gift from Prof. Xiaoyong Zhang, the Department of Infectious Medicine, Nanfang Hospital, China) and horseradish peroxidase-conjugated goat anti-rabbit IgG (Zhongshan Golden Bridge Biotechnology Co., Beijing, China) were incubated as described previously.

### Construction and Identification of CHB Model in Mice

**Figure 2:**
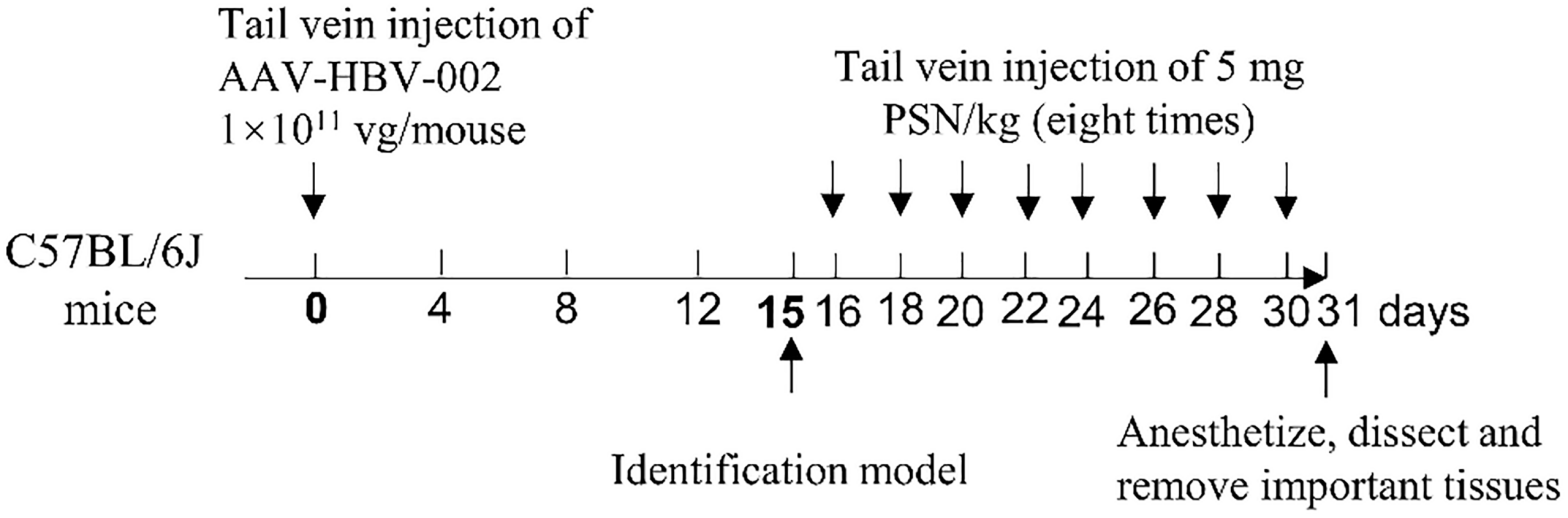
Schematic diagram of mouse modelling and PSN administration.

The recombinant adeno-associated virus AAV-HBV-002 applied to construct the hepatitis B model was purchased from PackGene Biotech, Co., Ltd. AAV-HBV-002 containing 1.3× HBV genome, is characterized by the production of HBV DNA, HBeAg and HBsAg, and the genotype is C2 and serotype is adr. Sixteen mice were each injected with AAV-HBV-002 into the tail vein at a dose of 1×10^11^ vg. Another group of eight mice was injected with an equal volume of PBS as normal controls. Every four days, the mice’s mental condition, nutrition, and water consumption were assessed, and their body weight was measured and recorded. During collection every four days, a total of nearly 500 μL of orbital blood were collected and stored at -80°C. On fifteenth day, the expression of HBV DNA, HBeAg and HBsAg in serum was measured to determine whether the model had been successfully constructed. **Figure 2** depicts the process of constructing a hepatitis B model in mice by injecting virus into the tail vein.

### *In Vivo* Efficacy and Safety Trials of PSN

Fresh mouse blood was taken to prepare 2% erythrocyte suspension, to which different concentrations of PSN were added, and those with distilled water were used as the positive control group. After incubating at 37°C for 3 h, the colour of the supernatant was observed to monitor whether haemolysis had occurred. The 16 of mice who successfully constructed were randomly divided into PSN group and hepatitis B model group, and 8 untreated normal mice were selected as blank control group. The mice in the PSN group were given PSN and the other two groups were given saline in equal doses. In the PSN group, the effective dose of 5 mg/kg siRNA was administered every other day for 15 days, for a total of 8 doses. After completion of administration, the mice were anesthetized with 5% anhydrous ether, dissected, and perfused with PBS, then 1.5-2.0 mL of blood was collected. qPCR was used to detect the relative expression of HBV DNA (24). The primers and probe sequences were shown in **Table S1**. The levels of antigens HBeAg and HBsAg, transaminases of ALT and AST were detected by ELISA kits (NanJing JianCheng Bioengineering Institute). The expression of IFN-α, TNF-α, and IL-6 was detected by RT-qPCR and ELISA kits. The important organs of the mouse, such as heart, liver, kidney, spleen, lung and brain, were removed and fixed in 4% paraformaldehyde solution, before being embedded, sectioned, and HE staining. The procedure for administration of PSN intervention to mice is shown in **Figure 2**.

### *In Vivo* Targeting Experiments for PSN

Six of 6-8 weeks C57BL/6J male mice were selected to construct a mouse hepatitis B virus model. After successful modelling, the mice were randomly divided into two groups with 3 mice in each group, and the experimental and control groups were treated with PSN-Cy7 and SN-Cy7, respectively, with a tail vein injection dose of 5 mg/kg. The fluorescence intensity in liver, kidney, lung, spleen and brain tissues was observed by a multi-modal small animal live imaging system at 1 and 3 h after injection.

### Statistical Analysis

All data reported are representative of at least three independent experiments. The experimental results were statistically evaluated using SPSS v19.0 software and were reported as mean ± standard deviation (Mean ± SD). Comparisons between two groups of data were made using the t-test for two independent samples, and the Shapior-Wilk test for normality and chi-square test was used between multiple groups of data, with the test level α = 0.05 (two-sided), and *P*<0.05 was considered a statistically significant difference.

## Results

### Optimal Inhibitory Effect of siRNA-X on HBV DNA and HBsAg

**Figure 3:**
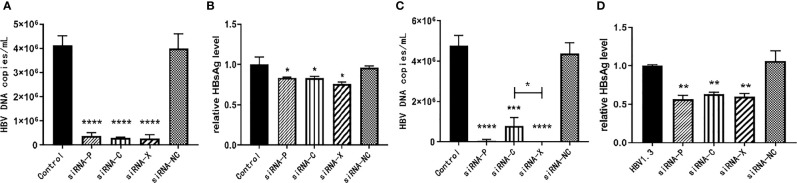
*In vitro* inhibition of HBV by siRNA. **(A, B)** pHBV1.3-HepG2 cells; **(C, D)** HepG2.2.15 cells. Data are represented as the mean ± SD from at least three independent experiments. *P < 0.05, **P < 0.01, ***P < 0.001, ****P < 0.0001.

The pHBV1.3-HepG2 and HepG2.2.15 cells were inoculated into six-well plates and the experiment was divided into five groups: PBS negative control group, siRNA-P, siRNA-C, siRNA-X, and siRNA-NC. As demonstrated in **Figure 3**, three of the siRNAs reduced HBV expression with statistically significant differences. For the inhibition of HBV DNA in the supernatant of HepG2.2.15 cells, siRNA-X was more effective than siRNA-C; for the inhibition of HBsAg, siRNA-X was the most effective (**Figures 3A–D**).

### PSN is Stable

**Figure 4:**
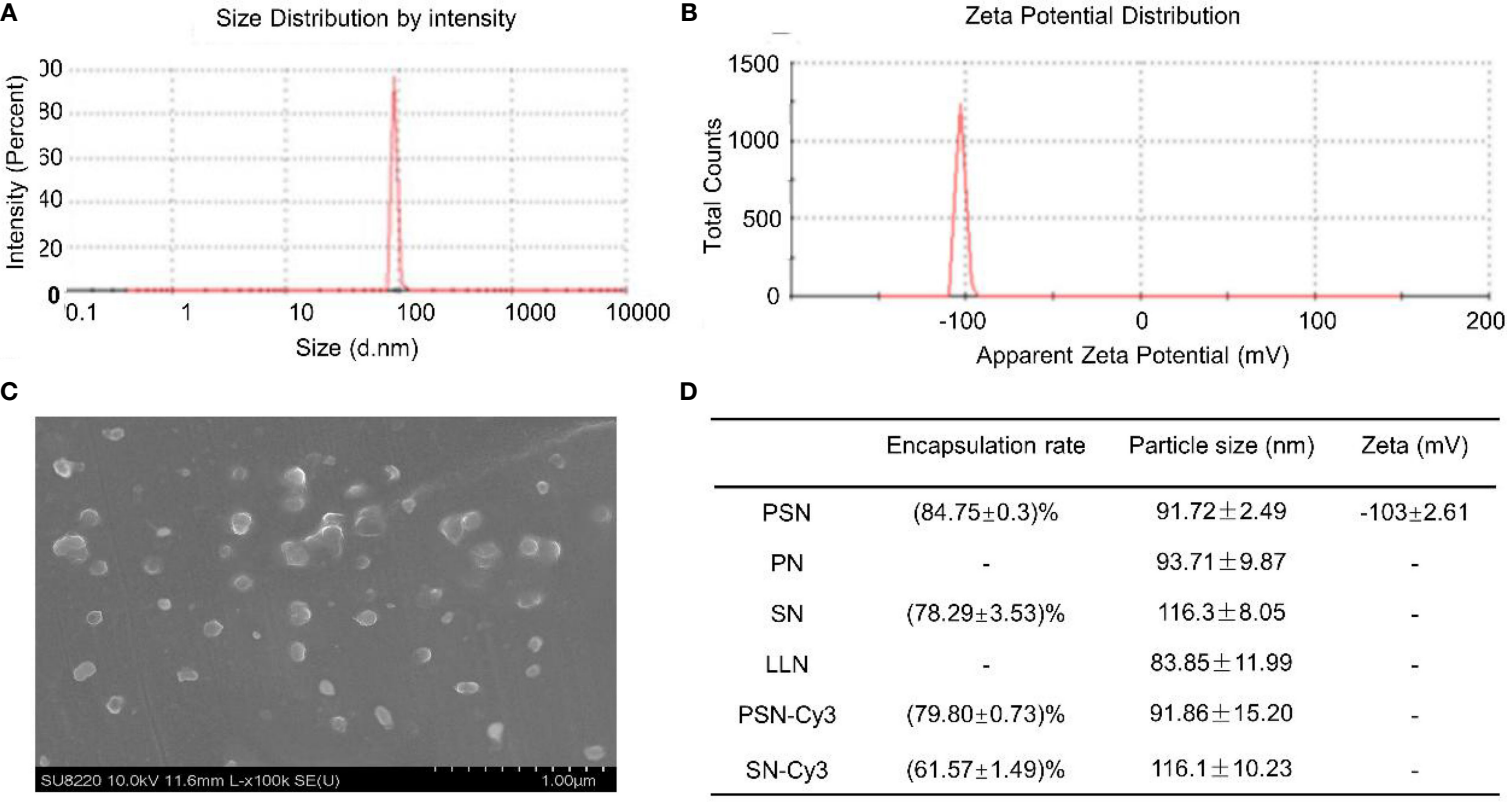
Properties of liposomal nanoparticles. **(A)** Particle size of PSN. **(B)** Zeta potential of PSN. **(C)** The ultrastructural morphology of PSN detected by scanning electron microscope. **(D)** Data on encapsulation rates, particle sizes, and zeta potentials of liposomal nanoparticles.

The particle sizes of nanoparticles ranged from (83.85 ± 11.99) nm to (116.30 ± 8.05) nm (**Figures 4A, D**). The zeta potential of PSN was (-103.01 ± 2.61) mV at 37°C (**Figure 4B**). Under the scanning electron microscope, the PSNs were spherical, with smooth surface and uniform size distribution, and the diameters were mostly about 100 nm (**Figure 4C**). The siRNA concentration in the filtrate before and after encapsulation was measured to calculate the encapsulation rate. The results showed that PSN achieved an encapsulation rate of (84.75 ± 0.30) %, which was higher than the others. The encapsulation rates and particle sizes of the nanoparticles are shown in **Figure 4D**.

**Figure 5:**
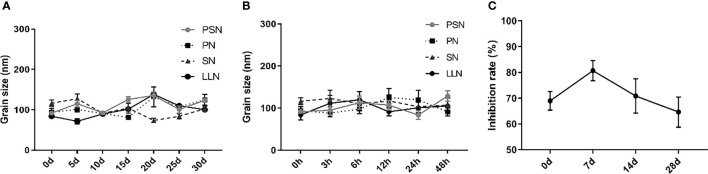
The stability of liposomal nanoparticles. **(A)** Temporal stability. **(B)** Stability in serum. **(C)** PSN activity at different time points.

Liposomal nanoparticles were stored at 4°C and particle size was tested every 5 days. There were some fluctuations in particle size, but no fragmentation or fusion into large particles (**Figure 5A**). It was also mixed with 10% serum and incubated at 37°C for 48 h to test the stability in serum, showing little fluctuation in particle size and no fragmentation or fusion into large particles (**Figure 5B**). HepG2.2.15 cells were treated with PSN stored at 4°C for different periods of time, showing that more than 60% of HBV DNA inhibition was retained for at least 28 days, and the activity was relatively stable (**Figure 5C**).

### PSN is Safe and Effective for Cells

We examined the CC50 and EC50 of PSN against three cell models and calculated the TI (CC50/EC50). The results (**Table 2**) showed that the TIs of PSN to three kinds of cells were close to or exceeded 100, indicating that PSN can specifically inhibit HBV with less damage to host cells and maintain good cell safety. RAW264 cells treated with 25 μg/mL PSN for 12 h did not cause significant changes in the expression of cytokines IFN-α, TNF-α, and IL-6, either in the cells or culture supernatants (**Figure S1**).

**Table 2:** The cytotoxicity, efficacy, and therapeutic index of liposomal nanoparticles.

	CC50 (μg/mL)	EC50 (μg/mL)	TI
pHBV1.3-HepG2	338.95 ± 5.48	3.19 ± 0.02	121.10
HepG2.2.15	450.11 ± 13.96	2.80 ± 0.26	160.81
HepG2-N6	273.34 ± 12.01	3.06 ± 0.02	97.66

### PSN Precisely Targets NTCP Receptors

**Figure 6:**
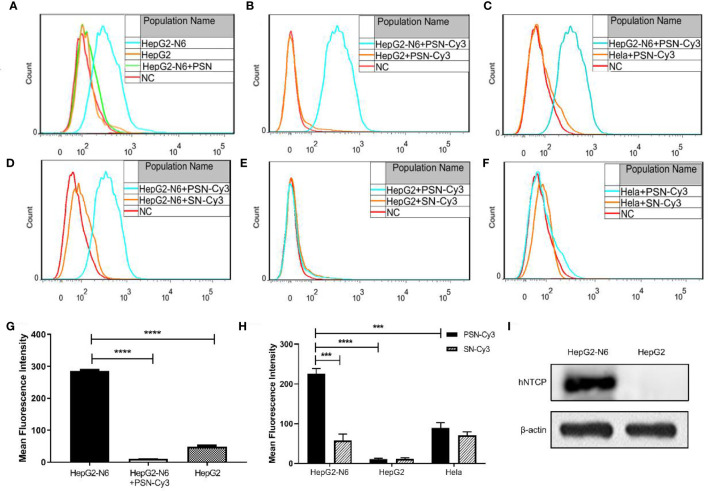
Targeting detection of liposomal nanoparticles. **(A)** After NTCP-FITC antibody labeling, flow cytometry detects NTCP receptor expression on cells and PSN competition for receptor binding, NC is the same amount of cells stained with FITC-labeled isotype control antibody; **(B–F)** Flow cytometric detection of the targeting properties of siRNA-Cy3 loaded nanoparticles PSN and SN to HepG2-N6, HepG2 and Hela cells, NC is the corresponding cell added with the same amount of PBS; **(G)** Comparison of the fluorescence intensity of each treatment group in **Figure A**; **(H)** Comparison of fluorescence intensity of each experimental group in **Figure B**–**F**; **(I)** Western blot analysis of NTCP expression in HepG2-N6 and HepG2 cells. Data are represented as the mean ± SD from at least three independent experiments. ***P < 0.001, ****P < 0.0001.

After labeling with NTCP-FITC antibody, the expression of NTCP on the surface of each cell was detected by flow cytometry. The results (**Figure 6A**) showed that the fluorescence intensity of HepG2-N6 cells was high, while HepG2-N6 cells was significantly lower after PSN treatment with statistically difference (*P*<0.0001) (**Figure 6G**). The fluorescence intensity of HepG2 cells incubated with NTCP-FITC antibody was lower (*P*<0.0001) (**Figure 6G**), and the fluorescence intensity of HepG2 cells did not change significantly after PSN treatment. The same results were obtained by fluorescence microscopy, as detailed in **Figures S2A–C**. The results indicate that HepG2-N6 cells have a high level of NTCP receptors on surface, and PSN specifically binds to NTCP and induces a reduction in its endocytosis. And the results (**Figure 6I**) by western blot analysis further indicated that HepG2-N6 cells have a high level of NTCP receptors.

The Cy3-labeled siRNA was used to quantify the targeting of PSN to various cells. Flow cytometry results showed that the fluorescence intensity of PSN-Cy3 in HepG2-N6 cells was significantly higher than that in HepG2 cells as well as Hela cells (*P*<0.0001, *P*<0.001) (**Figures 6B, C, H**); the fluorescence intensity in PSN-Cy3-treated HepG2-N6 cells was significantly higher than that in SN-Cy3 group (*P*<0.001) (**Figures 6D, H**); while the fluorescence intensity in PSN-Cy3-treated HepG2 cells and Hela cells had almost the same fluorescence intensity as the SN-Cy3 group (**Figures 6E, F**). Under the fluorescence microscope, PSN-Cy3 showed the highest brightness in HepG2-N6 cells, but weak fluorescent signal in HepG2 cells and Hela cells (**Figures S2D–I**). The results indicate that PSN has strong NTCP binding ability and can be endocytosed by HepG2-N6 cells with high NTCP expression, but less by HepG2 cells and Hela cells with low expression of NTCP receptor.

### PSN Strongly Inhibits Intracellular HBV

The inhibitory effect of liposomal nanoparticles on HBV was examined using HepG2.2.15 cells. Except for LLN, all liposomal nanoparticles had a certain inhibitory effect on HBV with statistically significant difference. The inhibition rate of PSN on pgRNA and HBV DNA was stronger, reaching 95.81% and 83.53%, respectively, and on HBxAg, cccDNA, HBeAg, and HBsAg was around 40% (**Figures S3A–F**). The inhibitory impact of PSN on HBV was further investigated using HepG2 cells transfected with pHBV1.3 plasmid as a model of HBV-infected cells, which showed similarly result (**Figures S3G–L**).

**Figure 7:**
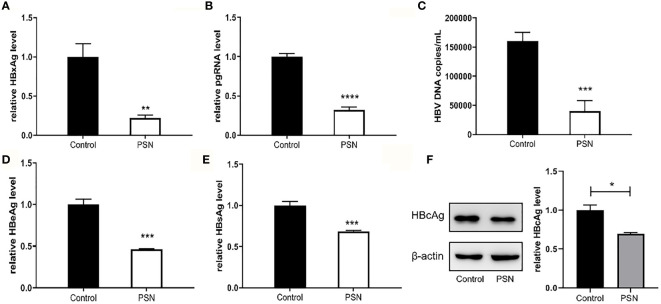
Inhibitory effect of nanoparticles on HBV, when nanoparticles and HBV simultaneously act on HepG2-N6. **(A, B)** Inhibitory effect of nanoparticles on HBxAg mRNA and pgRNA in cells. **(C–E)** Inhibitory effect of nanoparticles on HBV DNA, HBeAg and HBsAg in supernatant. **(F)** Western blot analysis of HBcAg expression in HepG2-N6 cells infected HBV, and expression after PSN treatment. Data are represented as the mean ± SD from at least three independent experiments. *P < 0.05, **P < 0.01, ***P < 0.001, ****P < 0.0001.

In addition, we detected HBV core antigen in HepG2-N6 cells infected with HBV by western blot, and the results (**Figure 7F**) showed that the core antigen in HBV-infected HepG2-N6 cells was significantly reduced after PSN treatment. We also tested the anti-HBV effect of PSN using HepG2-N6 cells before, during and after HBV infection respectively, and found that adding PSN before or at the time of HBV infection had a better pgRNA, HBeAg inhibition effect than after HBV infection, and the results were statistically different (**Figures 7**, **S4**). It showed that PreS/2-21 on the surface of nanoparticles could compete with HBV to bind NTCP receptors and function as a certain HBV entry inhibitor.

### PSN is Biocompatible without Hemolysis and Tissue Damage

**Figure 8:**
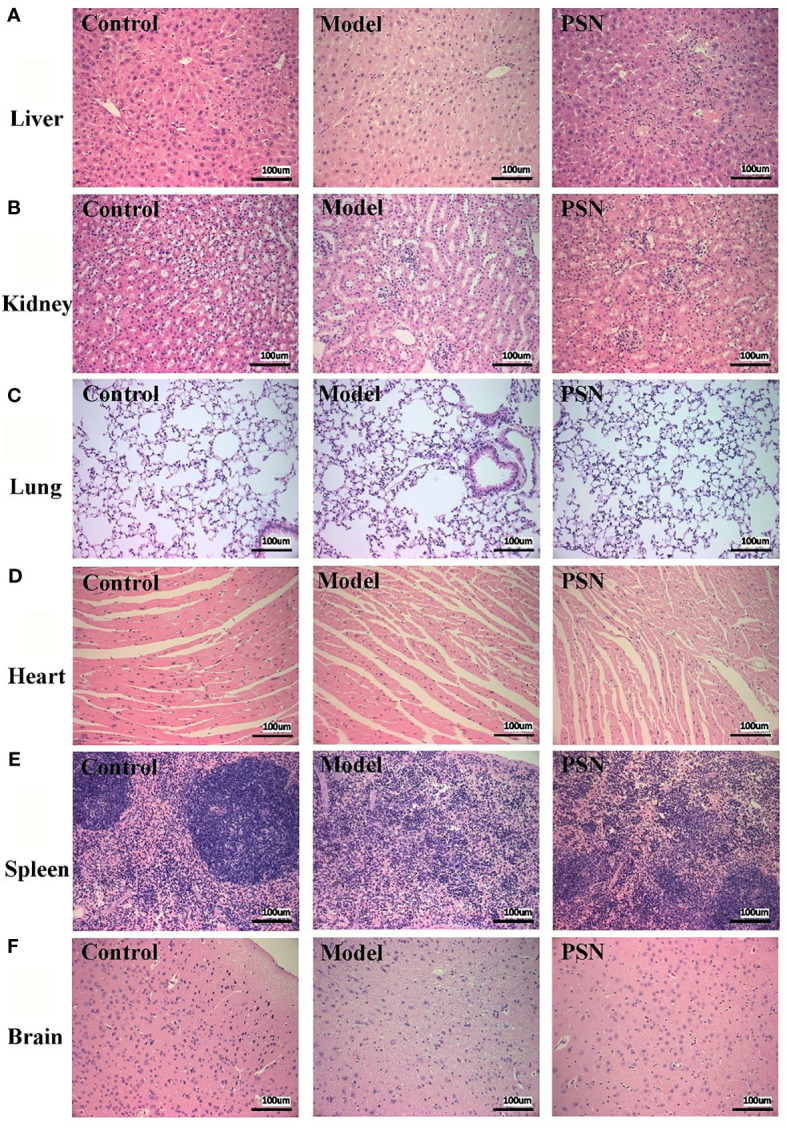
HE staining of mice's tissue sections in control, HBV model, and PSN groups (×200).** (A–F)** Liver, kidney, lung, heart, spleen, and brain tissue in sequence.

The results of the haemolytic assay showed no haemolysis in the PSN group, with all red blood cells sinking and the upper layer being a slightly cloudy yellow solution. In contrast, the distilled water group showed haemolysis and the solution was red (**Figure S5**). After 15 days of PSN treatment (5 mg/kg, qod, 8 time), all mice were anaesthetized and dissected, and pathological sections were taken from liver, lung, heart, kidney, spleen and brain tissues for HE staining. During modeling and administration, the mental status and body weight of the mice did not fluctuate apparently (**Figure S6**). The hepatocytes in the blank control group were normal in structure, with the nucleus in the centre of the cell and normal in size (left side of **Figure 8A**). The liver tissue of the mice in the hepatitis B model group was diffusely edema, the liver cells were obviously enlarged with indistinct boundaries, and there were a large number of vacuoles (middle of **Figure 8A**). The hepatocytes in the PSN group had clear borders and no evident pathological changes were observed (right side of **Figure 8A**). The rest of the tissues in the hepatitis B model group and the PSN groups showed no abnormalities (**Figures 8B–F**).

### PSN Reduces the Hepatitis B Viral Load *In Vivo*

**Figure 9:**
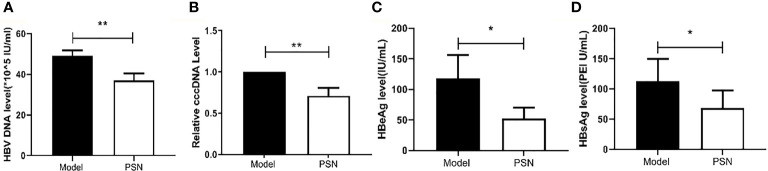
Inhibition of HBV by PSN in mice. **(A–D)** qPCR or ELISA tests for the expression of HBV DNA, cccDNA, HBeAg and HBsAg in mice's serum. Data are representative of three independent experiments with n = 8 mice per group. *P < 0.05, **P < 0.01.

After successful modelling, the experimental group was given PSN intervention for 15 days, while a model control group and a blank control group were set up and given an equal volume of saline. After 15 days of treatment, a high copy of HBV DNA could be detected in the hepatitis B model group at a concentration of (49.13 ± 2.68)×10^5^ IU/mL. In the PSN group, the level of HBV DNA was lower than that in the hepatitis B model group, at (37.13 ± 3.35)×10^5^ IU/mL (**Figure 9A**), with an inhibition rate of 24.43%. The relative expression of cccDNA was also lower in the PSN group compared to the hepatitis B model group (**Figure 9B**), and the inhibition rate of cccDNA by PSN was 28.93%. After PSN intervention, the expression of HBeAg decreased from (117.97 ± 38.14) PEI U/mL to (52.56 ± 17.55) PEI U/mL and the expression of HBsAg decreased from (112.38 ± 37.61) IU/mL to (68.79 ± 28.90) IU/mL with statistically significant difference (**Figures 9C, D**), and the inhibition rates of PSN on HBeAg and HBsAg were 55.45% and 38.79%, respectively.

### PSN Reduces the Levels of Transaminases and Cytokines *in Vivo*

**Figure 10:**
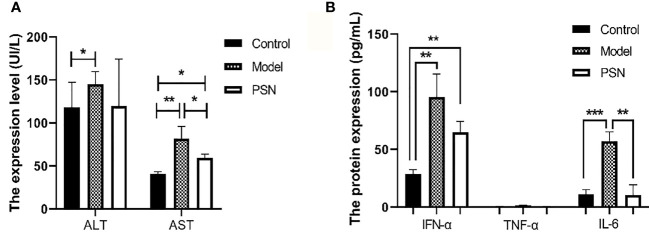
ELISA tests for the expression of transaminases and cytokines in mices's serum after PSN treatment. **(A, B)** The relative expression of transaminases ALT and AST, cytokines IFN-α, TNF-α, and IL-6. Data are representative of three independent experiments with n = 8 mice per group. *P < 0.05, **P < 0.01, ***P < 0.001.

After 15 days of PSN treatment, the expression of ALT and AST were lower than those in the hepatitis B model group and close to those in the blank control group (**Figure 10A**). The mRNA expression of cytokines IFN-α, TNF-α and IL-6 was significantly reduced after PSN treatment, which appeared to be closer to the levels of the control group (**Figure S7**). Compared with the model group, the protein expression of IL-6 and IFN-α in the serum of mice in the PSN group decreased, among which IL-6 decreased with statistical difference (*P* < 0.01), while the expression level of TNF-α appeared to be relatively low among the three groups (**Figure 10B**).

### PSN Targets Liver Tissue *In Vivo*

**Figure 11:**
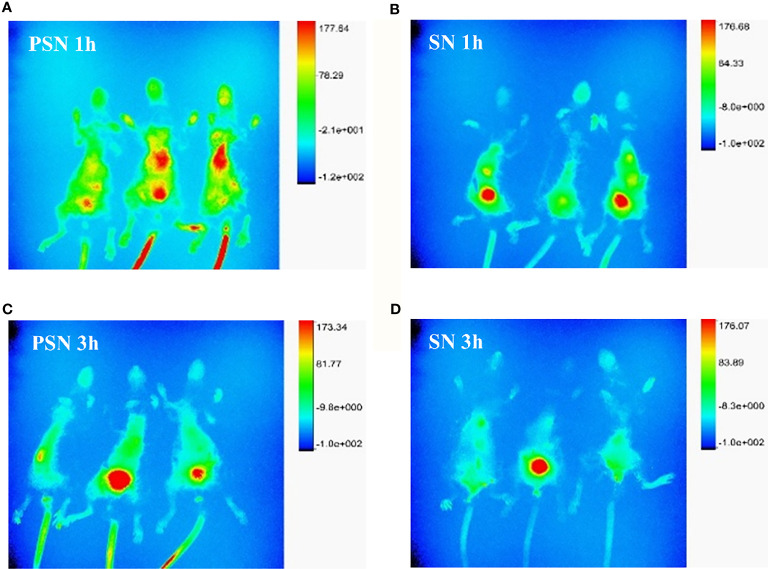
Fluorescence distribution of live and *in vivo* mice tissues. **(A, B)**. Fluorescence distribution of 1 h *in vivo* imaging of mice after PSN-Cy7 and SN-Cy7 injection respectively; **(C, D)**. Fluorescence distribution of 3 h *in vivo* imaging of mice after PSN-Cy7 and SN-Cy7 injection respectively.

After 1 h of injection of PSN-Cy7 and SN-Cy7, *in vivo* imaging of mice revealed that the fluorescence in the PSN group was concentrated in the liver and gastrointestinal tract, while in the SN group was concentrated in the gastrointestinal tract with only weak fluorescence in the liver (**Figures 11A, B**). After 3 h of injection, the fluorescence in the PSN group was concentrated in the gastrointestinal tract, while some mice in the control group only had fluorescence in the gastrointestinal tract or even no fluorescence in the body (**Figures 11C, D**).

## Discussion

The HBV genome is a 3.2 kb partial double-stranded circular DNA that can be transcribed into 3.5, 2.4, 2.1, and 0.7 kb viral transcripts containing four open reading frames (S, C, P, and X, respectively) (25). All HBV transcripts are encoded in an overlapping reading format with the same polyadenylation signal (PAS) and a shared 3’ terminus (26). Among them, the gene X encodes HBx, which is a 17 kDa protein. In addition to acting as a trans-activating transcription factor for oncogenes, it is also a protein necessary for virus replication. It initiates and maintains the transcription of cccDNA template, which has an impact on apoptosis and metabolism. These effects may lead to chronic HBV infection (27, 28). And as the HBx sequence is expressed in all four viral mRNAs, it appears to be an attractive target for anti-HBV siRNA development (25). In contrast, several studies have shown that not only viral mRNA expression but also HBV replication were inhibited in cultured cells and in mice by using siRNAs targeting the P, S, C, and X genes (29–32). We tested siRNAs targeting different genes of HBV by cellular assays, siRNA-X showed the best inhibitory effect on HBV (**Figure 3**). Therefore, we chose to construct targeting nanoparticles on the basis of siRNA-X. The liposomal nanoparticles have the characteristics of safety, stability, efficiency, ease of targeting modifications, and the possibility of long circulation, which make them ideal as carriers for the delivery of gene drugs *in vivo*. Two kinds of siRNA drugs are currently on the market, Onpattro (patisiran) (33) and Givlaari (givosiran) (34), both encapsulated in liposomes, the former targeting transthyretin (TTR) as an injectable treatment for hereditary transforming amyloidosis (hATTR) (35), and the latter targeting a neurotoxic intermediate (ALAS1) for the treatment of acute hepatic porphyria (36). In view of this, and based on the important role that the PreS1 protein plays in HBV infection, PEG was used in this study to improve the stability of liposomes, and PreS/2-21 was added to the liposome surface to achieve active targeting, i.e., to reduce drug accumulation in normal tissues and increase drug concentrations in liver tissue.

In this study, PreS/2-21 modified siRNA nanoparticles were investigated *in vivo* and *in vitro* experiments, and were validated in terms of safety, targeting and antiviral effects, all of which achieved the expected results and were consistent with theory. In addition, PN only blocking extracellular HBV entry and SN only inhibiting intracellular HBV replication were designed as controls. Compared with PN and SN, PSN inhibited pgRNA, HBxAg, and HBeAg with significantly increased efficiency, indicating that nanoparticles modified with PreS/2-21 specifically inhibit viral infection through competitive binding; while the nanoparticles loaded with siRNA specifically inhibit intracellular viral replication. The nanoparticles with the modification of PreS/2-21 on the surface, along with internal loading of siRNA, showed the highest anti-HBV activity. Such a combination design is quite beneficial to improve the drug effect. Also, PreS/2-21 has been proven to be an excellent HBV entry inhibitor, which can effectively inhibit HBV infection of cells (37). In this study, PreS/2-21 was coupled to the surface of nanoparticles, hoping that it would confer targeting ability to hepatocytes while also preventing HBV infection. NTCP is a receptor protein for HBV infection that is extensively expressed on the surface of hepatocytes. We confirmed that the NTCP receptor was abundantly expressed on the surface of HepG2-N6 cells by fluorescent antibody labeling, flow cytometry and western blot, and that PSN could compete with anti-NTCP-FITC antibody to bind NTCP receptor, initially confirming the targeting of NTCP by PSN. The siRNA in the nanoparticles was subsequently labelled with the fluorescent dye of Cy3 and detected by fluorescence microscopy and flow cytometry, demonstrating that PSN-Cy3 could be enriched in HepG2-N6 cells, while the fluorescent signal was weak in HepG2 cells and Hela cells with low NTCP expression, indicating that PSN has a strong targeting ability. Cytotoxicity assays further confirm that PSN specifically inhibits only viral replication, with TIs approaching or exceeding 100, without causing cytolysis and significant changes in cytokines, all of which indicate a high safety for PSN. In addition, the particle size of PSN does not change significantly with the addition of serum or temporarily storage, and PSN retains more than 60% of cellular activity even after one month of storage, indicating that PSN is stable and has excellent drug-forming properties.

Based on the *in vitro* experiments, this study further investigated the safety and efficacy of PSN through *in vivo* experiments. The hepatitis B model mice were successfully constructed and treated with PSN for 15 days, during which time the mental status and body weight of the mice did not change appreciably. The results of HE staining of the heart, liver, spleen, kidney, brain and lung sections from the PSN-treated mice showed that PSN intervention could improve liver injury in HBV-infected mice without other organ damage, which indicated that the efficacy and safety of PSN were not in doubt. For the antiviral effect of PSN, we found that the levels of HBV DNA, HBeAg and HBsAg in the PSN-treated hepatitis B mice group were significantly lower than those in the model group, which is consistent with the results of related studies that siRNA can significantly reduce the levels of HBeAg and HBsAg *in vivo* (7, 38). Furthermore, we discovered that PSN lowered ALT and AST levels in hepatitis B mice, confirming the antiviral action of PSN *in vivo* and improving liver function to some extent. The mice were then administered targeted PSN and non-targeted SN interventions and performed *in vivo* imaging after 1 and 3 h of the intervention in both groups. Thus, it was confirmed that PSN is equally liver tissue-targeting *in vivo*.

It is clear that the safety, targeting and anti-HBV efficacy of PSN have been validated and confirmed by *in vitro* and *in vivo* trials, which provides new ideas for the development of hepatitis B drugs and offers hope for the eradication of hepatitis B. This is the significance of this study. The two therapeutic agents currently approved for the treatment of CHB in adults are interferon-alpha (Peg-IFNα) and NAs: nucleosides (lamivudine, telbivudine, and entecavir) and nucleotides (adefovir and tenofovir). Although NAs are well tolerated and can suppress viral replication to undetectable levels, the long-term treatment required to maintain viralogical control frequently leads to drug resistance or serious side effects such as nephrotoxicity, reduced bone mass, or bone marrow failure. Although Peg-IFNα has a high probability of clearing HBsAg, it has restricted by expensive cost, a slew of adverse effects, or low response rates (39). The combination of Peg-IFNα and NAs improves functional cure rates, but it is only appropriate for a limited proportion of patients (40). The PSN is modified on the basis of RNA interference, and siRNA is encapsulated by liposomal nanoparticles coupled with PreS/2-21 to boost its stability and targetability. PreS/2-21 inhibits viral infection outside hepatocytes by blocking receptors, and RNAi inhibits viral replication in hepatocytes by mediating gene silencing. Thus, PSN has the advantages of siRNA antiviral agent, which reduces all measurable viral products as well as actively targets the hepatocyte NTCP receptor. Therefore, PSN is a drug with great potential for the treatment of hepatitis B.

Of course, the present study has certain limitations due to time restriction. On the one hand, we only selected one siRNA as the encapsulated drug for research *in vitro* and *in vivo*, and the experimental results showed that the encapsulated drug played a crucial role in inhibiting HBV, but the encapsulated drug was not limited to just one, which suggested that we could select multiple gene therapeutic drugs and co-package them in liposomal nanoparticles to play a combined role but without interaction, thereby improving the inhibitory effect on HBV and contributing to the cure of acute and chronic hepatitis B. On the other hand, the NTCP receptor discoverer Li (41) further found that the mouse NTCP (mNTCP) was unable to support HBV infection, however, it can bind to pre-S1 of HBV L protein and is functional in transporting substrate taurocholate. When mNTCP residues 84 to 87 were substituted by human counterparts, mNTCP can effectively support viral infections, suggesting that 84–87 aa residues are determinants of NTCP’s function as an HBV entry receptor (41). Although the reason why mNTCP does not support HBV infection remains to be further elucidated, mNTCP supports the specific binding of the pre-S1 lipopeptide on the cell surface, despite the binding ability of mNTCP to the pre-S1 region appears to be weaker than that of hNTCP. From this point of view, the *in vivo* targeting experiment in mice we designed is theoretically feasible. HBV binding to mNTCP may be insufficient and additional molecules or mechanisms are required to trigger the following early infection process (42). The fact that the liposome fuses with the cell membrane to deliver the drug into the cell compensates for the above deficiency, so PSN theoretically has the ability to mimic early infection. Importantly, the results in **Figures 8A** and **11A** are consistent with theory, showing that PSN indeed targets and enters mouse hepatocytes to exert inhibitory activity. Admittedly, the AAV-HBV-mouse model is not a natural infection process, whether it can form true cccDNA was still controversial. It is necessary to further study on the inhibitory effect of PSN on HBV through the natural infection model of hepatitis B virus constructed by human hepatocyte chimeric mice. In conclusion, PreS/2-21-directed nanoparticles loaded with HBV gene therapy drugs are expected to be promising for the treatment of CHB.

## Conclusion

SiRNA nanoparticles guided by PreS/2-21 can target and inhibit the infection and replication of hepatitis B virus, which has been confirmed at the level of animal experiments. Compared with the conventional drugs used in clinical practice, it shows obvious advantages.This study also provides a new idea for the treatment of chronic hepatitis B.

## Data Availability Statement

The original contributions presented in the study are included in the article/**Supplementary Material**. Further inquiries can be directed to the corresponding author.

## Ethics Statement

The animal study was reviewed and approved by Ethics Committee of Southern Medical University.

## Author Contributions

WM and TL conceived and designed the study. ZL, YD, JL, LY, and JW conducted the experiments. JF and HF analyzed the data. LG and JY wrote the paper. All authors reviewed and edited the manuscript. All authors contributed to the article and approved the submitted version.

## Funding

This work was supported by the Natural Science Foundation of Guangdong Province (grant no. 2021A1515011828 and no. 2022A1515010985). 

## Conflict of Interest

The authors declare that the research was conducted in the absence of any commercial or financial relationships that could be construed as a potential conflict of interest.

## Publisher’s Note

All claims expressed in this article are solely those of the authors and do not necessarily represent those of their affiliated organizations, or those of the publisher, the editors and the reviewers. Any product that may be evaluated in this article, or claim that may be made by its manufacturer, is not guaranteed or endorsed by the publisher.
