# Supplementary material for: PreS/2-21-Guided siRNA Nanoparticles Target to Inhibit Hepatitis B Virus Infection and Replication
Source: Front Immunol. 2022 Apr 29;13:856463. doi: 10.3389/fimmu.2022.856463 (PMC9098953; doi:10.3389/fimmu.2022.856463)
Supplement: Supplementary file 1 [file DataSheet_1.pdf]

Supplementary Information

PreS/2-21-guided siRNA Nanoparticles Target to Inhibit Hepatitis B Virus Infection and Replication

Lixia Gao[1†], Jie Yang[1†], Jutao Feng[2†], Ziying Liu[3], Ying Dong[4], Jiangyan Luo[1], Liangzhentian Yu[1], Jiamei Wang[1], Hongying Fan[1], Weifeng Ma[1*] and Tiancai Liu[4*]

1 Department of Microbiology, School of Public Health, Southern Medical University, Guangzhou, 510515, PR China,

2 Department of Hepatobiliary Surgery, The First Affiliated Hospital of Guangzhou Medical University, Guangzhou, 510120, PR China,

3State Key Laboratory of Organ Failure Research, Guangdong Provincial Key Laboratory of Viral H epatitis Research, Department of Infectious Diseases, Nanfang Hospital, Southern Medical Universit y, Guangzhou, 510515, PR China,

4 Institute of Antibody Engineering, School of Laboratory Medicine & Biotechnology, Southern Medical University, Guangzhou, 510515, PR China.

*Corresponding Author: Tiancai Liu; Weifeng Ma

E-mail: liutc@smu.edu.cn; maweifeng919@163.com

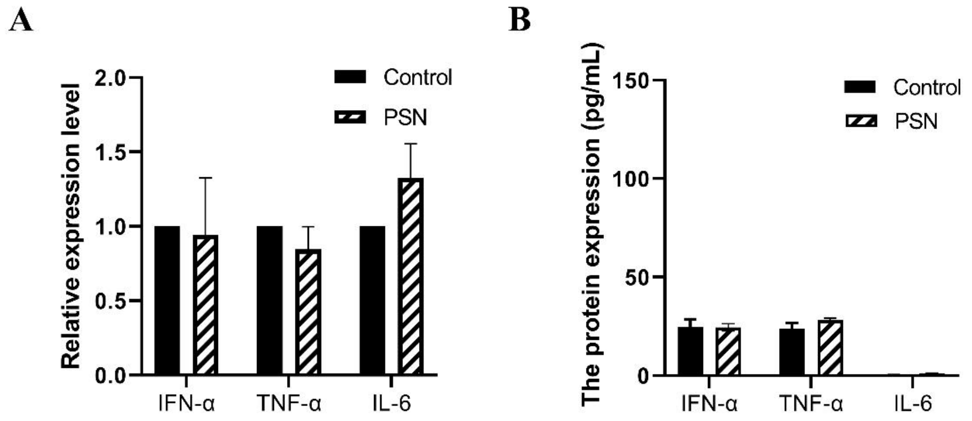

Figure S1. RT-qPCR **(A)** and ELISA tests **(B)** for the expression of cytokines IFN-α, TNF-α, and IL-6 in RAW264.7 cells after 25 μg/mL PSN treatment. Data are represented as the mean ± SD from at least three independent experiments.

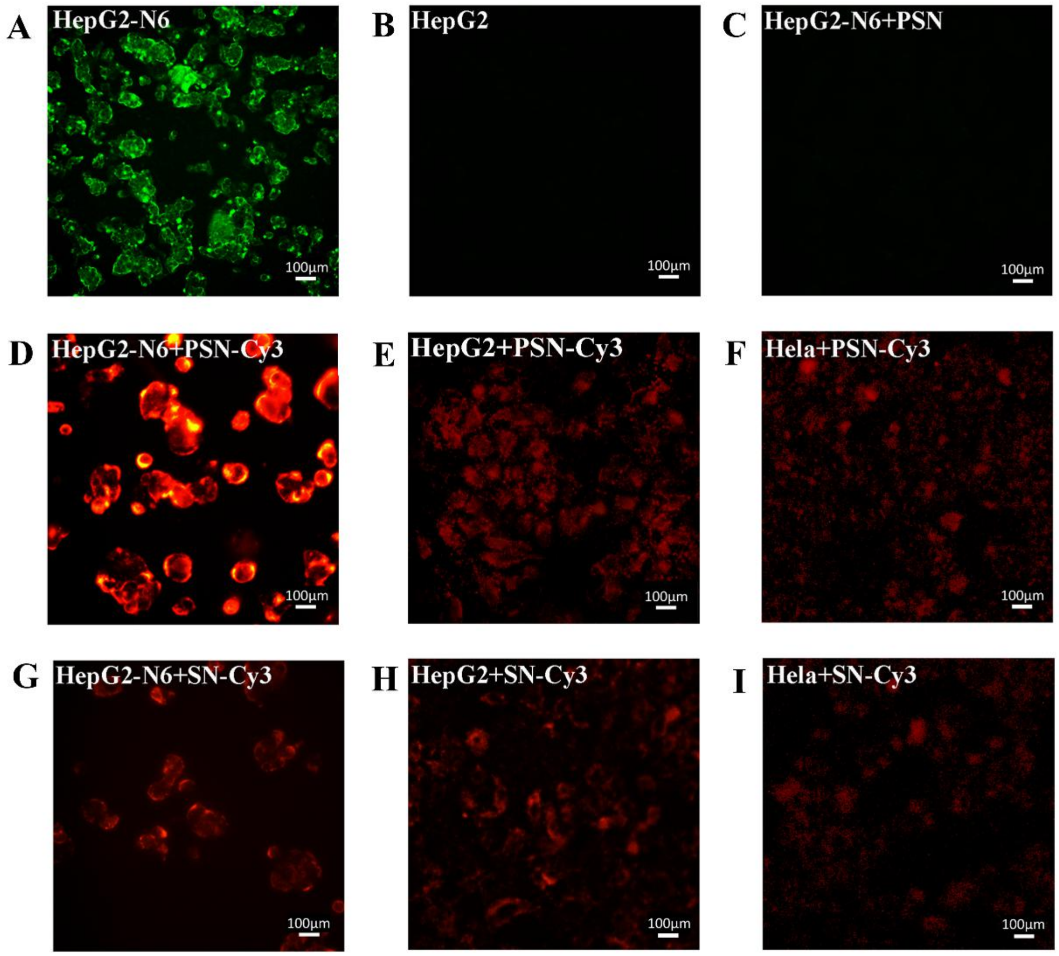

Figure S2. Targeting detection of liposome nanoparticles

(A-C). After NTCP-FITC antibody labeling, fluorescence microscopy detection of the expression of NTCP on cells and PSN competition for receptor binding; **(D-I)**. Fluorescence microscopy detection of the targeting properties of siRNA-Cy3 loaded nanoparticles PSN and SN to HepG2-N6, HepG2 and Hela cells.

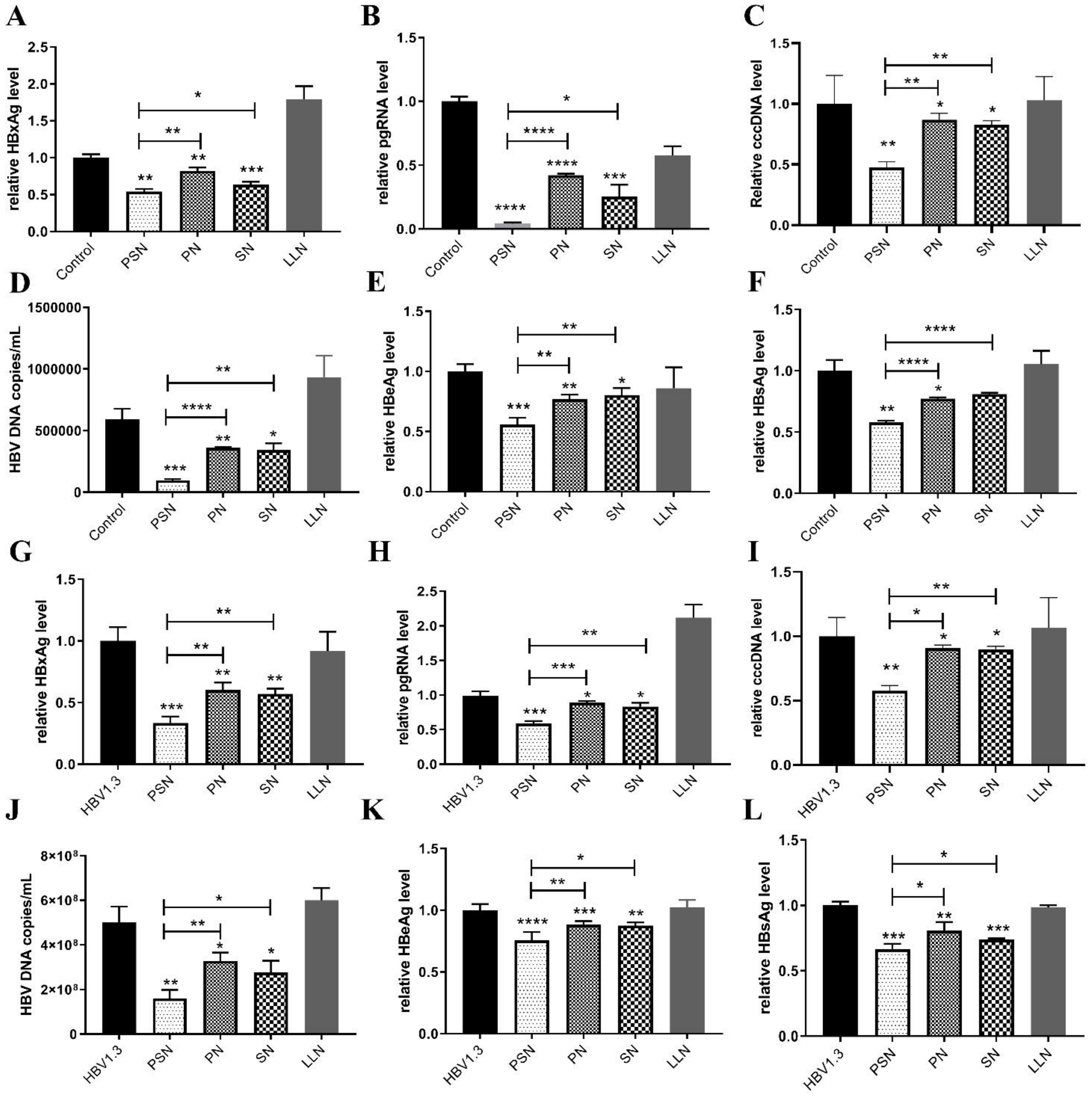

Figure S3. Effect of liposomal nanoparticles on the inhibition of HBV in HepG2.2.15 cells and pHBV1.3-HepG2. **(A-C)**. Inhibitory effect of liposomal nanoparticles on HBxAg mRNA, pgRNA and cccDNA in HepG2.2.15 cells. **(D-F)**. Inhibitory effect of liposomal nanoparticles on HBV DNA, HBeAg and HBsAg in supernatant of HepG2.2.15 cells. **(G-I)**. Inhibitory effect of liposomal nanoparticles on HBxAg mRNA, pgRNA and cccDNA in pHBV1.3-HepG2 cells. **(J-L)**. Inhibitory effect of liposomal nanoparticles on HBV DNA, HBeAg, and HBsAg in supernatant of pHBV1.3-

HepG2 cells. Data are represented as the mean ± SD from at least three independent experiments. **P*<0.05, ***P*<0.01, ****P*<0.001, *****P*<0.0001.

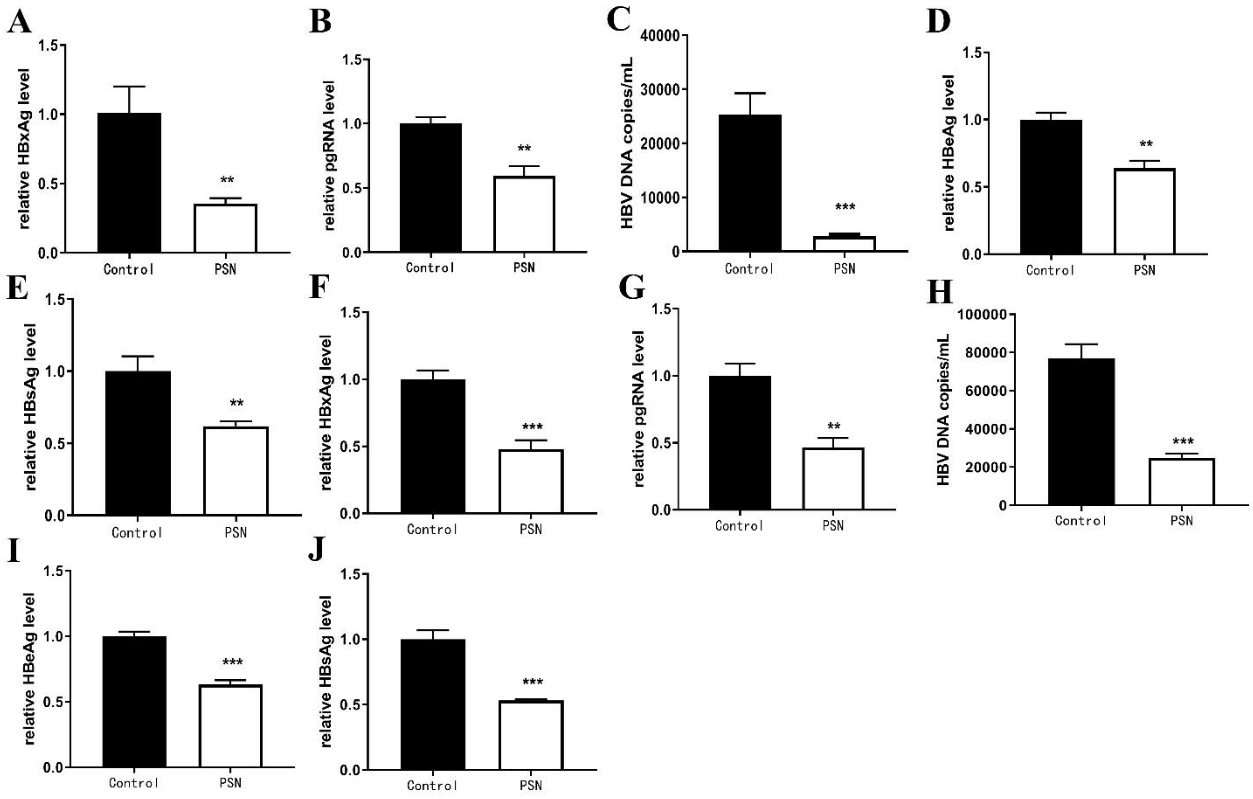

Figure S4. PSN inhibition of HBxAg, pgRNA, HBV DNA, HBeAg and HBsAg in HepG2-N6 cells before **(A-E)** and after HBV infection **(F-J)**. Data are represented as the mean ± SD from at least three independent experiments. ***P*<0.01, ****P*<0.001.

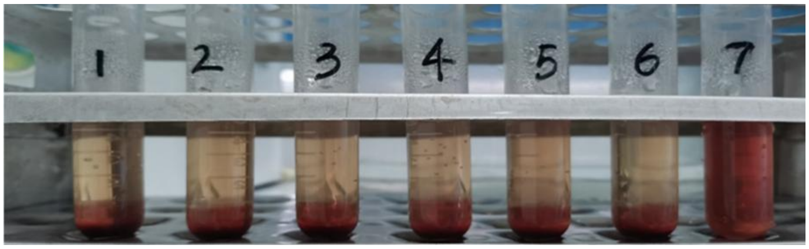

Figure S5. The results of the haemolytic assay. Tubes 1-6 are the erythrocyte suspension with 0, 0.1, 0.2, 0.4, 0.8, 1.6 mg/mL PSN, respectively; tube 7 is the erythrocyte suspension with distilled water.

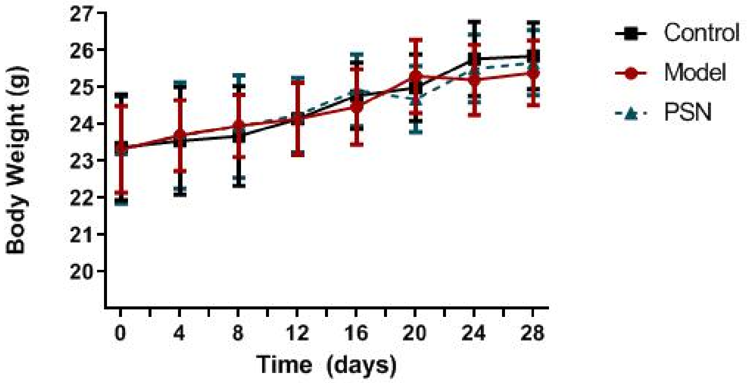

Figure S6. Average body weight during the modeling of hepatitis B mice and administration.

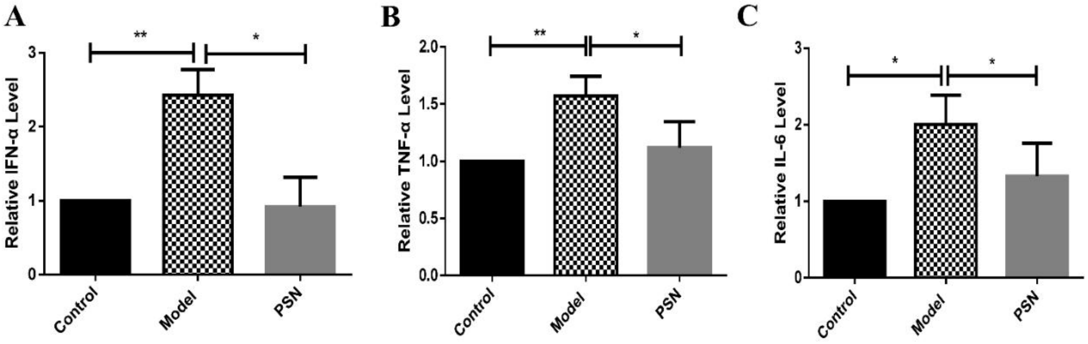

Figure S7. RT-qPCR analysis for the expression of cytokines IFN-α **(A)**, TNF-α **(B)**, and IL-6 **(C)** in mices's serum after PSN treatment. Data are representative of three independent experiments with n = 8 mice per group. **P*<0.05, ***P*<0.01.

Table S1. Primers for qPCR or RT-qPCR

Primers	Primer sequences
HBxAg-F	GTCTGTGCCTTCTCATCTGCC
HBxAg-R	CTCAAGGTCGGTCGTTGACA
pgRNA-F	GGAGTGCGAATCCACACTC
pgRNA-R	AGAAGAACTCCCTCGCCTC
HBV-F	ATACTGCACTCAGGCAAGC
HBV-R	GCCTCGTCGTCTAACAAC
GAPDH-F	GAAGGTGAAGGTCGGAGT
GAPDH-R	CATGGGTGGAATCATATTGGAA
cccHBV-1519-F25	5'-ACGGGGCGCACCTCTCTTTACGCGG-3'
cccHBV-1886-R25	5'-CAAGGCACAGCTTGGAGGCTTGAAC-3'
cccHBV-1685-F20FM	5′-(FAM)-AACGACCGACCTTGAGGCAT-(MGB)-3′
IL-6 F	CTGCAAGAGACTTCCATCCAG
IL-6 R	AGTGGTATAGACAGGTCTGTTGG
TNF-α F	CCGCGACGTGGAACTGG
TNF-α R	GGCCATTTGGGAACTTCTCAT
IFN-α F	GAGGCCGTGCTGGTGCTCA
IFN-α R	TGATTTCTGCTCTGACAACCTCCC
